# Caffeic acid phenethyl ester inhibits the growth of bladder carcinoma cells by upregulating growth differentiation factor 15

**DOI:** 10.1016/j.bj.2021.10.006

**Published:** 2021-10-15

**Authors:** Chen-Pang Hou, Ke-Hung Tsui, Kang-Shuo Chang, Hsin-Ching Sung, Shu-Yuan Hsu, Yu-Hsiang Lin, Pei-Shan Yang, Chien-Lun Chen, Tsui-Hsia Feng, Horng-Heng Juang

**Affiliations:** a Department of Urology, Chang Gung Memorial Hospital at Linkou, Taoyuan, Taiwan; b Graduate Institute of Clinical Medical Science, College of Medicine, Chang Gung University, Taoyuan, Taiwan; c Department of Urology, Shuang Ho Hospital, New Taipei City, Taiwan; d Department of Medicine; TMU Research Center of Urology and Kindey, College of Medicine, Taipei Medical University, Taipei, Taiwan; e Department of Anatomy, College of Medicine, Chang Gung University, Taoyuan, Taiwan; f Graduate Institute of Biomedical Sciences, College of Medicine, Chang Gung University, Taoyuan, Taiwan; g School of Nursing, College of Medicine, Chang Gung University, Taoyuan, Taiwan

**Keywords:** Bladder, GDF15, CAPE, AMPK, MAPK, NDRG1

## Abstract

**Background:**

Caffeic acid phenethyl ester (CAPE), a bioactive component of propolis, has beneficial effects on cancer prevention. Growth differentiation factor 15 (GDF15) is an antitumor gene of bladder cancer. Therefore, this study investigated the anti-cancer effect of CAPE on bladder carcinoma cells and related mechanisms.

**Methods:**

The expressions of GDF15, N-myc downstream-regulated gene 1 (NDRG1), and maspin, and the activations of extracellular signal regulated kinase (ERK), c-jun Nterminal kinase (JNK), p38, and 50 adenosine monophosphate-activated protein kinase (AMPK) α1/2 in human bladder cells after gene transfection or knockdown were determined by immunoblot, real-time reverse transcriptase-polymerase chain reaction (RT-qPCR), and reporter assays. The assays of 5-ethynyl-2′-deoxyuridine (EdU), CyQUANT cell proliferation, and Matrigel invasion, and the xenograft animal study were used to assess the cell proliferation, invasion, and tumorigenesis.

**Results:**

GDF15 expression in epithelial cells was negatively correlated with neoplasia *in vitro*. Also, GDF15 exhibits in bladder fibroblasts and smooth muscle cells. CAPE-induced expressions of NDRG1 and maspin decreased cell proliferation and invasion of bladder carcinoma cells in a GDF15-dependent manner *in vitro*. The xenograft animal study suggesting CAPE attenuated tumor growth *in vivo*. CAPE increased phosphorylation of ERK, JNK, p38, and AMPKα1/2 to modulate the GDF15 expressions. Pretreatments with ERK, JNK, or p38 inhibitors partially inhibited the CAPE effects on the inductions of GDF15, NDRG1, or maspin. Knockdown of AMPKα1/2 attenuated the CAPE-induced GDF15 expression and cell proliferation in bladder carcinoma cells.

**Conclusions:**

Our findings indicate that CAPE is a promising agent for anti-tumor growth in human bladder carcinoma cells *via* the upregulation of GDF15.

## Graphical abstract

**Figure:**
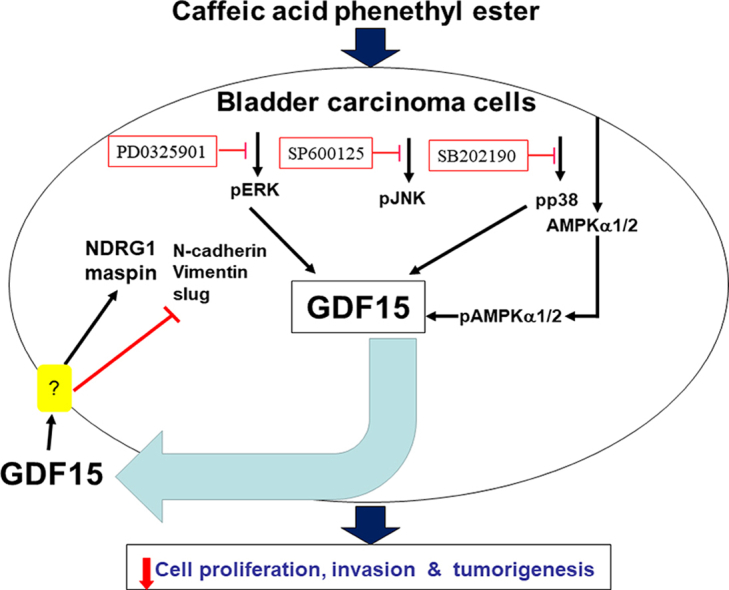

**At a glance of commentary**

**Scientific background on the subject**

Growth differentiation factor-15 (GDF15) enhances NDRG1 and maspin to downregulate cell proliferation and invasion of the bladder carcinoma cells. Caffeic acid phenethyl ester (CAPE), a bioactive compound from propolis, possesses antitumor effect and induces NDRG1 gene expression through the ERK signaling pathway to suppress the tumor growth in certain cancers.

**What this study adds to the field**

This study confirmed that CAPE attenuates proliferation, invasion, and growth of bladder carcinoma cells in vitro and in vivo, inducing the expressions of GDF15, NDRG1, and maspin via ERK, p38, or AMPKα1/2 signaling pathways, indicating that CAPE is a promising preventive agent in the tumor growth of human bladder carcinoma cells.

## Background

Growth differentiation factor-15 (GDF15) is a secretory dimeric protein with widely different functions in tissue-specific and cell-specific presentations in various cancers [[1], [2], [3]]. GDF15 strongly correlates with many health problems such as obesity, diabetes, cardiovascular disease, and cancers, hence, is a potential indicator of disease progression [4]. Prior studies have suggested that GDF15 is a tumor suppressor gene and upregulated by DNA demethylation and p53 in the human bladder cancer [5,6]. Previously, we reported that GDF15 enhanced the expressions of N-myc downstream-regulated gene 1 (NDRG1) and maspin to downregulate the cell proliferation and invasion in bladder carcinoma cells [7]. However, the regulation of GDF15 in the human bladder cells has not been well elucidated. Furthermore, GDF15 possesses cytokine characteristics, acting on the transforming growth factor-beta (TGF-β) receptor to modulate target genes [8,9]. Also, an orphan receptor in the glial cell-derived neurotrophic factor (GDNF) family, GDNF family receptor alpha-like (GFRAL), is a GDF15 receptor [10] with limited expression in a few specific organs, tissues, and cells [11].

Propolis, a natural product derived from honeybee hives, has a broad spectrum of pro-health properties such as anti-inflammatory, antimicrobial, antioxidative, antiulcer, hepatic- and cardio-protective, as well as antitumor. Propolis is therefore widely used in foods and beverages for health maintenance and promotion [12]. Caffeic acid phenethyl ester (CAPE) is the most bioactive propolis compound possessing effects of anti-oxidation, anti-angiogenesis, anti-inflammation, radical scavenger, and antitumor [13]. Studies have shown that CAPE represses several human cancers including oral, lung, breast, prostate, melanoma, head and neck squamous carcinoma, and nasopharyngeal carcinoma, etc. [[14], [15], [16], [17], [18], [19]]. Further, it has suggested that the antitumor effect of CAPE is relevant to the regulation of many biological processes such as cell cycle, expressions of NF-κB, Bak, Bcl-2-associated X protein, SKp2, p53, p21, and p27 genes, as well as Akt phosphorylation, caspase, matrix metalloproteinase, and mitogen-activated protein kinase (MAPK) pathways [20,21]. Our previous studies indicated that CAPE induced NDRG1 gene expression through the ERK signaling pathway to suppress the growth of oral (OSCC) and nasopharyngeal carcinoma (NPC) cells [18,19].

This study aimed to determine the antitumor effect of CAPE and evaluate the potential molecular mechanisms of CAPE on the expression of GDF15 and its downstream genes, NDRG1 and maspin, in human bladder carcinoma cells.

## Materials and methods

### Materials, cell lines, and cell culture

The bladder transitional cell carcinoma cell lines, RT-4, HT1376, TSGH-8301, and T24 cells, were purchased from the Bioresource Collection and Research Center (BCRC, Hsinchu, Taiwan) and cultured as described previously [22]. Primary bladder epithelial cells (HBdEC; ATCC PCS 420–010) were purchased from ATCC (Manassas, VA, USA). Human bladder stromal fibroblasts (HBdSF; SC-4330), human bladder smooth muscle cells (HBdSMC; SC-4310), smooth muscle cell medium (SMCM; SC-1101), and fibroblast medium (FM; SC-2301) were purchased from ScienCell (Carisbad, CA, USA). Fetal calf serum (FCS) was purchased from HyClone (Logan, UT, USA), RPMI 1640 media was obtained from Invitrogen (Carlsbad, CA, USA), and Matrigel was purchased from BD Biosciences (Bedford, MA, USA). CAPE was purchased from Selleckchem (Houston, TX, USA). ERK inhibitor (PD0325901) and p38 inhibitor (SB202190) were purchased from Sigma Aldrich Co. (St. Louis, MO, USA). c-jun Nterminal kinase (JNK) inhibitor II (SP600125) was purchased from Merck Millipore (Burlington, MA, USA).

### Gene knockdown

Cells were seeded into 6-well plates 1 day before the culture medium was replaced with RPMI-1640 medium plus 10% FCS and 5 μg/ml polybrene (Santa Cruz Biotechnology, Santa Cruz, CA, USA), then transduced with GDF15 (sc-39798-V) or AMP-activated kinase (AMPK) α1/2 (sc-45312-V) shRNA transduction particles (Santa Cruz Biotechnology). Two days after transduction, the cells were selected by incubation with 10 μg/ml puromycin dihydrochloride for at least five more generations. The mock-transfected cells were transduced with control shRNA transduction particles (sc-10808-v, Santa Cruz Biotechnology) and clonally selected in the same manner as the target gene knockdown cells.

### 5-ethynyl-20-deoxyuridine (EdU) flow cytometry assay

Cells were cultured in a serum-free medium for 24 h. After being incubated with or without CAPE for another 48 h in RPMI1640 medium with 10% serum, and EdU (5-ethynyl-2′-deoxyuridine; 10 μM) was added to the culture medium for 2 h. Subsequently, the cells were collected, fixed, and permeabilized using the Click-iT EdU Flow Cytometry Assay Kit (Thermo Fisher Scientific Inc. Waltham, MA, USA). The EdU fluorescence of the 5000–10,000 cells was detected using an Attune NxT acoustic focusing cytometer (Thermo Fisher Scientific Inc.).

### CyQUANT cell proliferation assay

Cell proliferation was measured by the CyQUANT cell proliferation assay kit (Invitrogen, Carlsbad, CA, USA). Briefly, 3000 cells were seeded into each well of a 96-well plate in RPMI 1640 medium with 10% FCS for 48 h. Cells were washed twice with phosphate-buffered saline (PBS) and the cell pellet was frozen at −80 °C for 1 h. After the cell pellet was thawed at room temperature, a 200 μl of CyQUANT GR dye with lysis buffer was added to each well and incubated for 10 min before the fluorescence was measured at 488 nm excitation using the synergy H1 microplate reader (BioTek Instruments, Inc., Beijing, China).

### Immunoblot assay

Equal amounts of whole cell lysates were separated on a 10–12% SDS-polyacrylamide gel. The blotting membranes were probed using the following antibodies: NDRG1 (42–6200; Invitrogen), SAPK/JNK (56G8, #9258, Cell Signaling, Danvers, MS, USA), phospho-SAPK/JNK (Thr183/Tyr185, 81E11, #8690, Cell Signaling), p44/42 MAPK (ERK 1/2; 137F5, #4695, Cell Signaling), phospho-p44/42 MAPK (ERK 1/2, Thr202/Tyr204, #9101, Cell Signaling), p38 MAPK (D13E1, #8690, Cell Signaling), phospho-p38 MAPK (Thr180/Tyr182, #9211, Cell Signaling), AMPKα1/2 (#5831, Cell signaling), phospho-AMPKα1/2 (Thr 172; #2535, Cell Signaling), GDF15 (Ab206414, Abcam, Cambridge, MA, USA), alpha-smooth muscle actin (α-SMA) (Ab5694, Abcam), uroplakin II (UPK2) (LS-C41569; LifeSpan BioSciences, Seattle, WA, USA), and β-actin (MAB1501, Merck Millipore, Burlington, MA, USA). Band intensities were detected by the Western lightning plus-ECL detection system (PerkinElmer Inc, Waltham, MA, USA), recorded using the LuminoGraph II (Atto Corporation, Tokyo, Japan), and analyzed using the GeneTool Program of the ChemiGenius (Syngene Cambridge, UK).

### Real-time reverse transcriptase-polymerase chain reaction (RT-qPCR)

Total RNA was isolated with Trizol reagent, and cDNA was synthesized using the superscript III preamplification system (Invitrogen). Real-time PCR (qPCR) was performed using the CFX Connect Real-Time PCR system (Bio-Rad Laboratories, Foster City, CA, USA) as described previously [7]. FAM dye-labeled TaqMan MGB probes and PCR primers for target genes were purchased from Applied Biosystems for human GDF15 (Hs00171132_m1), NDRG1 (Hs00608387_m1), NDRG2 (Hs0104515_m1), NDRG3 (Hs00259237_m1), maspin (Hs00985283_m1), AMPKα1 (Hs01562315_m1), AMPKα2 (Hs00178903_m1), and β-actin (Hs01060665_g1). The mean cycle threshold (C_t_) values for target genes were normalized against the β-actin control probe to calculate ΔC_t_ values. All reactions were performed in at least three independent experiments.

### GDF15 reporter vector construct

The DNA fragment containing the enhancer/promoter of the GDF15 gene (−2887 to +3) was synthesized from BAC clones (CTC-251H24; Invitrogen) by PCR with primers (5′-GGTACCGACAAGAGCAGATTCATCC-3′ and 5′-TACTCTCCTCCTCCCCTAAC-3′). The DNA fragment was cloned into the pGL3-basic vector (Promega BioScience) at the *Kpn I* and *Xma I* cutting sites as described previously [7].

### Transient transfection and reporter assay

HT1376 cells were seeded at a density of 10^4^ cells/well in a 24-well plate and allowed to grow for 24 h prior to transfection. Cells were transiently transfected with GDF15 reporter vectors using the X-tremeGene HP DNA transfection reagent (Roche Diagnostics GmbH, Anaheim, Germany) for another 24 h, then treated with various concentrations of CAPE as indicated for 24 h and washed twice in PBS. The reaction was terminated by adding 200 μl of Luciferase Cell Culture Lysis Reagent (Promega Biosciences) and luciferase activity was determined in relative light units (RLU) using the Synergy H1 microplate reader (BioTek, Beijing, China) and adjusted by the concentration of protein in the whole-cell extract.

### Enzyme-linked immunosorbent assay

Cells were incubated in a 0.5 ml RPMI 1640 medium supplemented with 10% FCS and with/without CAPE as indicated. The protein levels of the cell supernatants were measured by GDF15 enzyme-linked immunosorbent assay kit (Catalog #: DY957; R&D Systems, Inc., Minneapolis, MN, USA) per the manufacturer's protocol. The protein levels in each sample were adjusted by the concentration of protein in the whole-cell extract, which was measured using the bicinchoninic acid protein assay kit (Pierce Protein Research, Rockford, IL, USA) as described previously [23].

### Matrigel invasion assay

The invasion ability of cells was determined by the *in vitro* Matrigel invasion assay as described previously [7]. Cells that migrated to the other side of the transmembrane were fixed with 4% paraformaldehyde, then stained with 0.1% crystal violet solution for 30 min. The numbers of cells that invaded the Matrigel were recorded microscopically and defined using the Image J program.

### Xenograft animal study

These studies were approved by the Chang Gung University Animal Use and Care Committee (CGU108-080) and they were conducted in accordance with the United States National Institutes of Health Guide for the Care and Use of Laboratory Animals as described previously [7]. The cells were detached from cell flask through treatment with Versene solution (Gibco, Lifer Technologies, Grand Island, NY, USA), washed with RPMI 1640 medium with 10% FCS, then resuspended in a PBS solution. Four-week-old male nude mice (BALB/cAnN-Foxn1) were from the animal center of the National Science Council. After one week, they were anesthetized intraperitoneally with a mixture of Zoletil 50 and Xylazine in PBS solution, and HT1376 (5 × 10^6^ cell/100 μl) was injected subcutaneously into the lateral back wall of each mouse. When tumor volumes reached around 75 mm^3^ on day 13, the xenograft mice were randomly divided into two groups: the study group (CAPE group) and the control group (vehicle group). CAPE (10 mg/kg/day) was dissolved in DMSO (1:1000 in PBS) and intra-peritoneally injected once per day, 5 days/week as described previously [18]. The control group was treated with vehicle (0.1% DMSO in PBS) only. The tumor volumes were measured using Vernier calipers and were determined by the formula: Volume = Length x Width x Width/2. Mouse body weight was measured three times weekly. Serum samples were collected from experimental animals by cardiocentesis during scarifications. The tumors of xenograft animals were collected and digested with protein lysis buffer or Trizol reagent for further analysis of mRNA or protein expressions of target genes after mice were sacrificed.

### Statistical analysis

Results were expressed as the mean ± standard error (S.E.). Statistical significance was determined by one-way ANOVA and Student's *t*-tests with the SigmaStat program for Windows version 2.03 (SPSS Inc, Chicago, IL, USA). Multiple comparisons were conducted using ANOVA with Tukey's post hoc test. Asterisks indicate significant differences (∗∗*P* < 0.01, ∗*P* < 0.05) compared to the vehicle-treated group unless otherwise indicated.

## Results

### GDF15 expression in bladder smooth muscle cells, fibroblasts, normal epithelial cells, and carcinoma cells

**Fig. 1:**
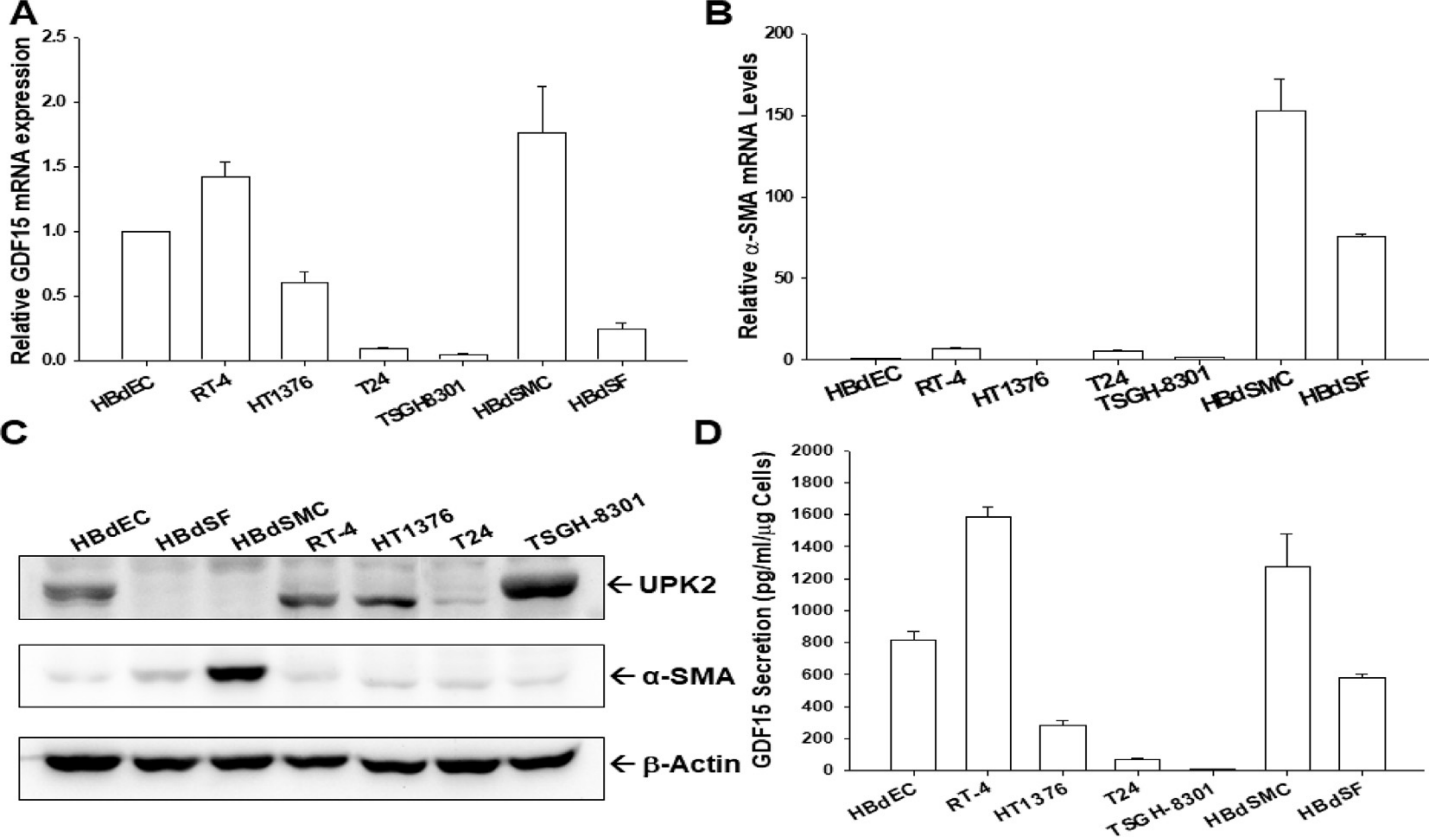
**Gene expression of GDF 15 in human bladder cells**. The mRNA levels of GDF15 (A) and α-SMA (B) in bladder smooth muscle cells (HBdSMC), fibroblast cells (HBdSF), normal epithelial cells (HBdEC), and carcinoma cell lines (RT-4, HT1376, T24, and TSGH-8301) were determined by RT-qPCR assays. Data are expressed as the mean ratio of mRNA in relation to HJBdEC cells (n = 3). (C) The protein levels of α-SMA, UPK2, and β-actin in bladder cells as indicated were determined by immunoblot assays. (D) GDF15 secretions from bladder cells as determined by ELISA (n = 4).

To understand the GDF15 expression in human bladder cells, the levels of GDF15, alpha-smooth muscle actin (α-SMA), and UPK2 were compared in human normal primary bladder epithelial cells (HBdEC) and stromal cells, i.e. bladder smooth muscle cells (HBdSMC) and bladder stromal fibroblasts (HBdSF), and four lines of cultured bladder carcinoma cells (RT-4, HT1376, T24, and TSGH-8301). The RT-qPCR analysis revealed that GDF15 was expressed in normal bladder epithelial cells and stromal cells with higher levels of GDF15 in HBdSMC and HBdEC, with RT-4 cells having the highest and TSGH-8301 cells having the lowest level of GDF15 among the carcinoma cells (Fig. 1A). Similar results were obtained with the ELISA assays (Fig. 1B). Further RT-qPCR and immunoblot assays showed that α-SMA was expressed only in bladder stromal cells (HBdSF and HBdSMC) with the highest expression in the HBdSMC (Fig. 1C). The bladder stromal cells did not express UPK2, a marker of bladder urothelial cells; however, UPK2 was expressed in normal bladder epithelial cells and bladder carcinoma cells, with the lowest expression in T24 cells (Fig. 1D).

### CAPE induces GDF15 expression and attenuates cell proliferation of bladder carcinoma cells

**Fig. 2:**
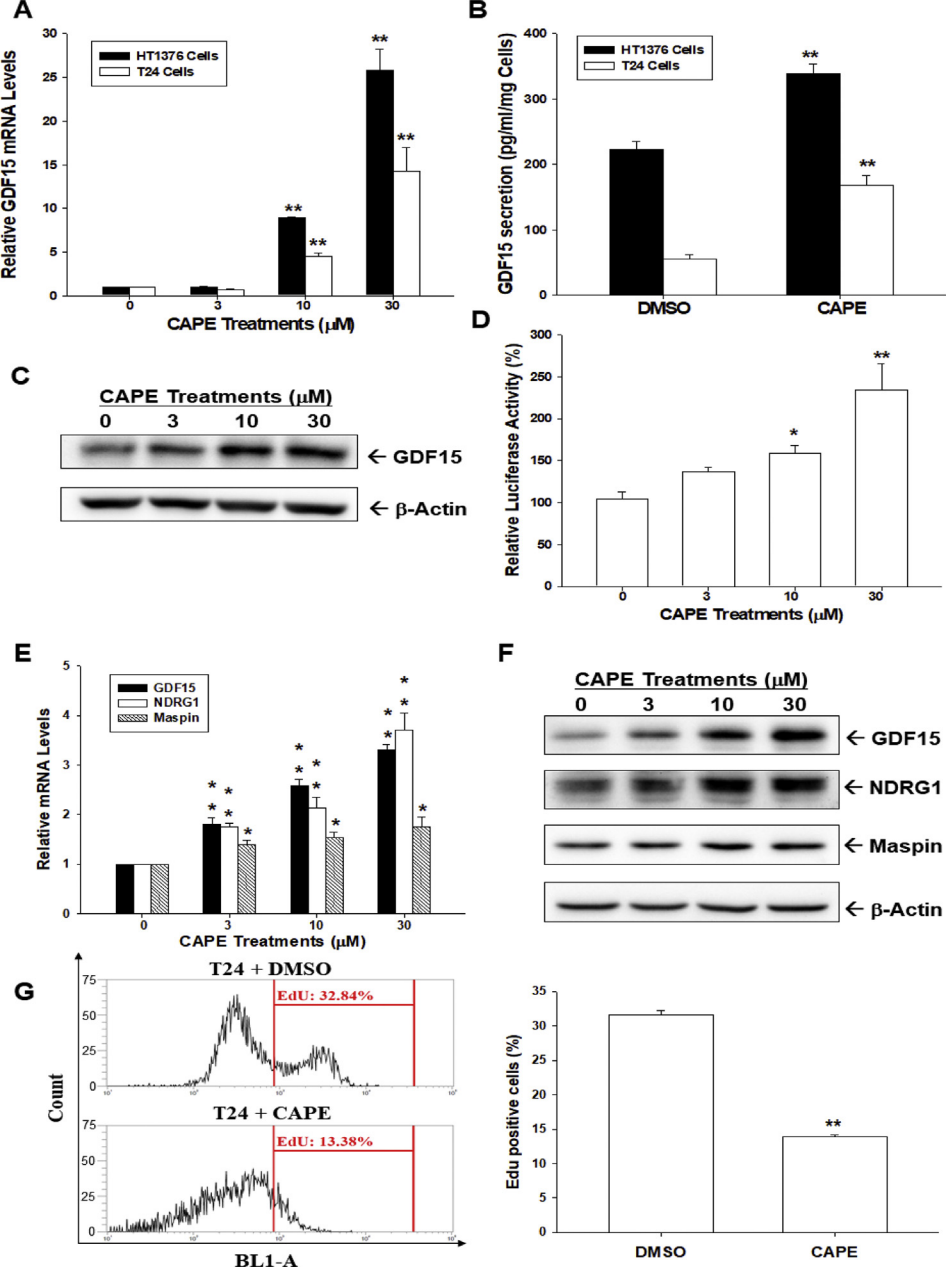
**Effects of CAPE on GDF15 expression and proliferation of bladder carcinoma cells**. (A) HT1376 and T24 cells were treated with various concentrations of CAPE as indicated for 24 h, then lysed, and the mRNA levels of GDF15 and β-actin were determined by RT-qPCR assays. Data are expressed as the mean ratio of mRNA relative to the control group (n = 3). (B) GDF15 protein levels in conditional media were determined by ELISA (n = 4). (C) T24 cells were treated with various concentrations of CAPE as indicated for 24 h and protein levels of GDF15 were determined by immunoblots. (D) The reporter activity of the GDF15 reporter vector in HT1376 cells treated with various dosages of CAPE for 24 h. Data are expressed as the mean percentage of luciferase activity relative to the mock-transfected group (n = 6). The mRNA (E) and protein (F) levels of target genes as indicated were determined by RT-qPCR and immunoblots. Data are expressed as the mean ratio of mRNA relative to the control group (n = 3). (G) Cell proliferation of T24 cells was determined by EdU assays after CAPE (30 μM) treatment for 24 h. Data are expressed as the mean percentage of Edu positive cells relative to the control group (DMSO-treated, n = 4). ∗∗*P* < 0.01, ∗*P* < 0.05.

CAPE induced GDF15 gene expression in a dose-dependent manner in both HT1376 and T24 cells (Fig. 2A), with the ELISAs confirming that CAPE (30 μM) treatment upregulated GDF15 secretion in both HT1376 and T24 cells (Fig. 2B). Immunoblot (Fig. 2C) and reporter assays with human GDF15 reporter vector (Fig. 2D) verified that CAPE upregulated GDF15 expression in T24 cells. Moreover, CAPE induced not only GDF15 but also NDRG1 and maspin expressions (Fig. 2E and F). EdU flow cytometry further revealed that CAPE downregulated the numbers of EdU incorporated cells by approximately 20% compared to vehicle control cells (Fig. 2G).

### Knockdown of GDF15 attenuates the antiproliferation and anti-invasion effects of CAPE

**Fig. 3:**
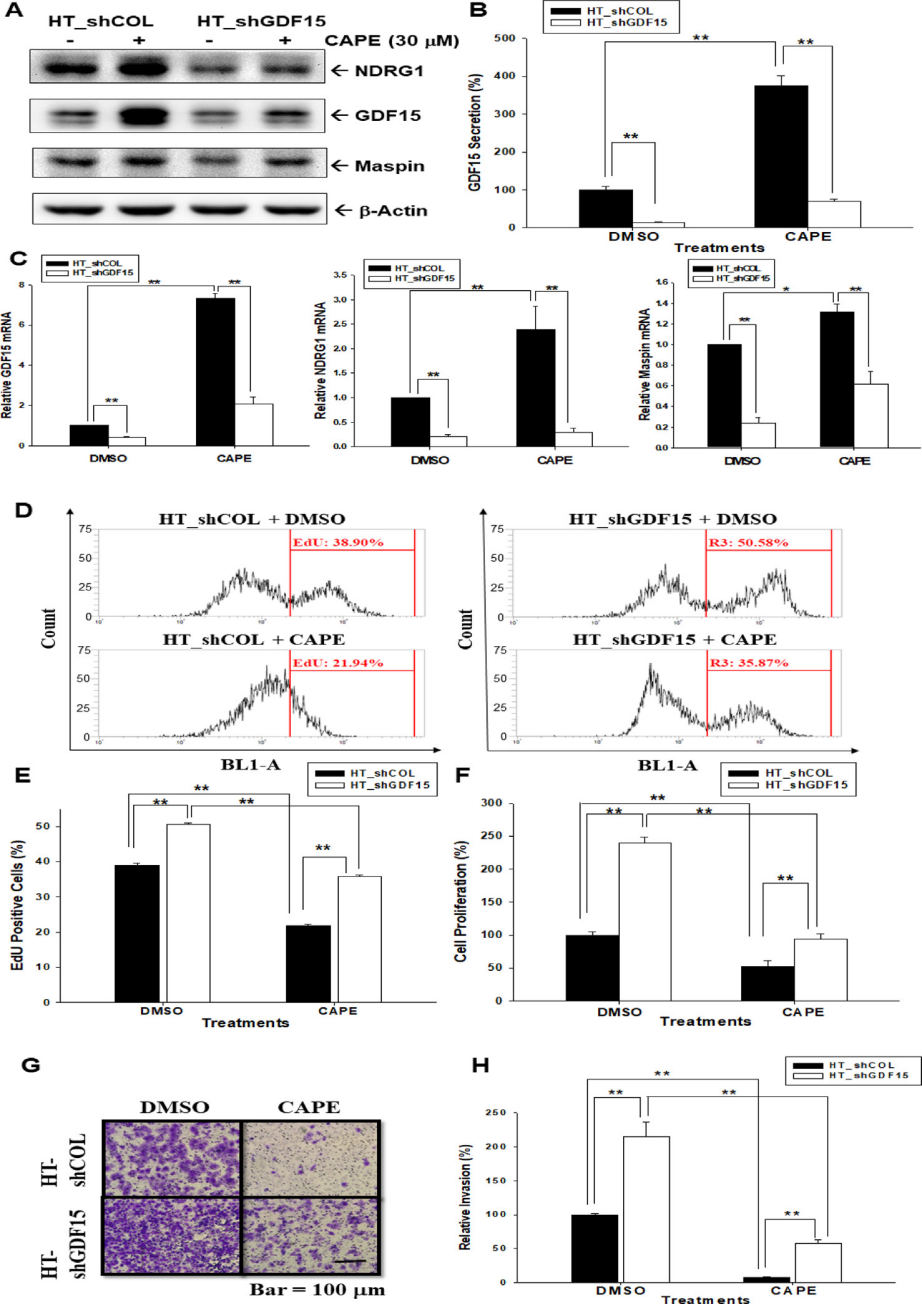
**Downregulation of cell proliferation and invasion by CAPE is GDF15-dependent in bladder carcinoma HT1376 cells**. (A) HT_shCOL and HT_shGDF15 cells were treated with (+) or without (−) CAPE (30 μM) for 24 h. The protein levels of target genes, as indicated, were determined via immunoblotting. (B) HT_shCOL and HT_shGDF15 cells were treated with (+) or without (−) CAPE (30 μM) for 24 h and GDF15 secretions in the supernatants of culture media were determined by ELISA (n = 4). (C) HT_shCOL and HT_shGDF15 cells were treated with (+) or without (−) CAPE (30 μM) for 24 h, then the relative mRNA levels of GDF15, NDRG1, and maspin were determined by RT-qPCR assays (n = 3). (D) Cell proliferation of HT_shCOL and HT_shGDF15 cells after treated with CAPE (30 μM) for 48 h was determined by EdU assays (n = 4). (E) The quantitative result of EdU assays. (F) HT_shCOL and HT_shGDF15 cells were treated with 30 μM CAPE for 48 h and cell proliferation was determined by the CyQuant assay. The data were presented as the mean percentage compared with the control group (DMSO-treated, n = 8). (G) HT_shCOL and HT_shGDF15 cells were treated with CAPE (30 μM) for 24 h and the cell invasive ability was measured by the Matrigel invasion assay after 24 h of incubation (H) The quantitative result of invasion assays. The data were presented as the mean percentage compared with the control group (DMSO-treated, n = 3). ∗∗*P* < 0.01, ∗*P* < 0.05.

To assess the effects of CAPE on the expressions of GDF15 and GDF15 downstream genes, NDRG1 and maspin, GDF15 was knocked down in HT1376 cells. Immunoblot assays confirmed that CAPE treatment (30 μM) induced GDF15, NDRG1, and maspin expressions, whereas GDF15-knockdown attenuated these effects (Fig. 3A). ELISAs verified that knockdown of GDF15 blocked the activation of CAPE on GDF15 secretion (Fig. 3B). As shown in Fig. 3C, GDF15-knockdown attenuated the effect of CAPE on the gene expressions of GDF15, NDRG1, and maspin, as well as enhancing the numbers of HT1376 cells stained with EdU by 11%. CAPE treatment (30 μM) downregulated EdU staining by 17% in HT_shCOL cells; however, knockdown of GDF15 attenuated the effect of CAPE on EdU staining in HT1376 cells (Fig. 3D and E). These results were confirmed by the CyQuant cell proliferation assay indicating that knockdown of GDF15 blocked the reduction effect of CAPE on cell proliferation (Fig. 3F). The Matrigel invasion assays (Fig. 3G and H) revealed that CAPE treatment (30 μM) downregulated cell invasion to 10% in HT1376 cells, whereas the invasion capacity of HT_shGDF15 cells was increased 2.2-fold compared to HT_shCOL cells. Moreover, GDF15-knockdown attenuated the effects of CAPE on reducing cell invasion in HT1376 cells.

### CAPE induces phosphorylation of ERK, p38, and JNK to upregulate GDF15, NDRG1, and maspin in bladder carcinoma HT1376 cells

**Fig. 4:**
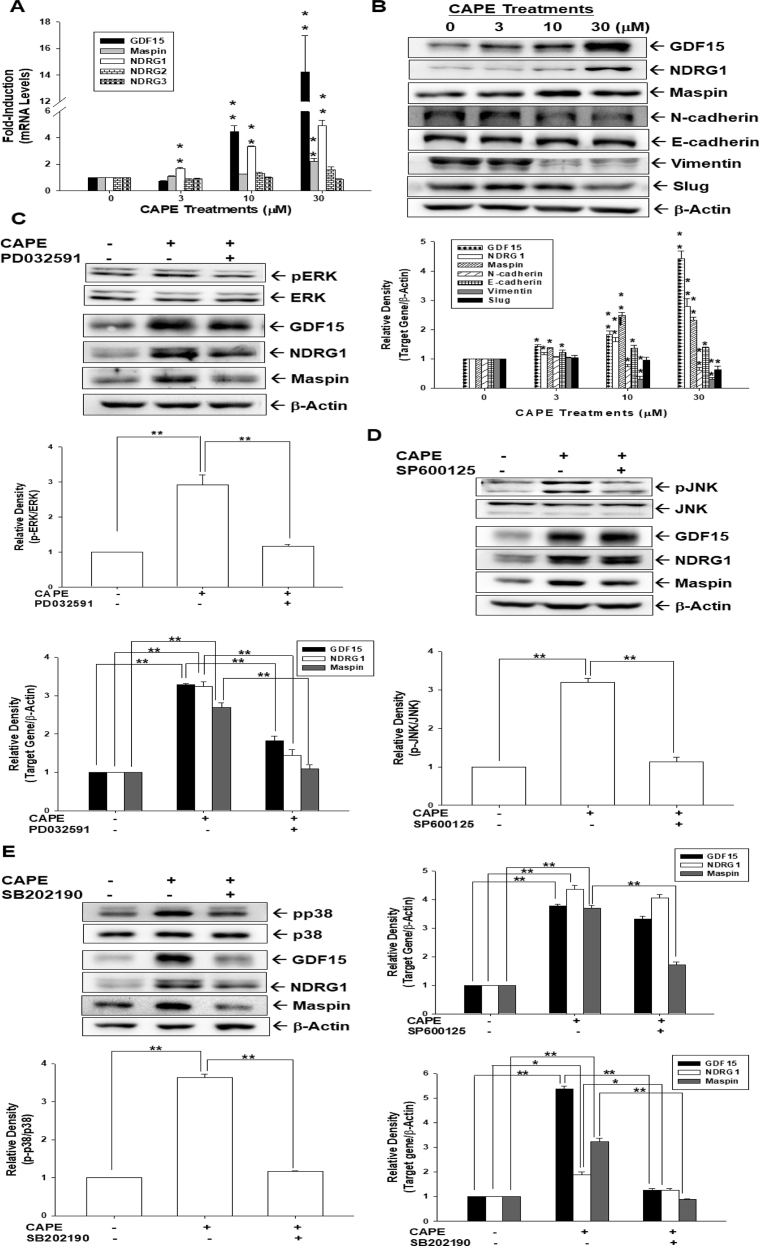
**CAPE induces the phosphorylation of ERK, JNK, and p38 to modulate the expressions of GDF15, NDRG1, and maspin in bladder carcinoma HT1376 cells**. (A) HT1376 cells were treated with various concentrations of CAPE as indicated for 24 h and the mRNA levels of target genes as indicated were determined by RT-qPCR assays. (B) Protein levels of target genes, as indicated, were determined by immunoblot assays after treatment with various concentrations of CAPE as indicated for 16 h in HT1376 cells. The quantitative data were expressed as the intensity of protein bands of the target genes/β-actin relative to the control solvent-treated group (n = 3). The expressions of ERK, p-ERK (C, top), JNK, p-JNK (D, top), p38, and p-p38 (E, top) were determined by the immunoblots after 20 min of 30 μM CAPE treatment with (+) or without (−) pretreatment of the indicated MAPK inhibitors for 1 h in HT1376 cells. The protein level of GDF15, NDRG1, maspin, and β-actin of HT1376 cells after CAPE treatment with (+) or without (−) pretreatment with PD0325901 (C, bottom), SP600125 (D, bottom), or SB202190 (E, bottom). The quantitative data were expressed as the intensity of protein bands of the p-target gene/target gene or target genes/β-actin relative to the control solvent-treated group (n = 3). ∗∗*P* < 0.01, ∗*P* < 0.05.

CAPE induced the gene expressions of GDF15, NDRG1, and maspin, but not NDRG2 and NDRG3 (Fig. 4A). Interestingly, the immunoblot assays further confirmed that CAPE treatment not only induced GDF15, NDRG1, and mapsin but also downregulated N-cadherin, vimentin, and slug while slightly increasing E-cadherin expression in HT1376 cells (Fig. 4B). To explore whether CAPE increased GDF15, NDRG1, and maspin expressions in bladder carcinoma cells through the MAPK pathway, HT1376 cells were pretreated with MAPK inhibitors, ERK (PD0325901), p38 (SB202190), or JNK (SP600125) for 1 h before exposure to CAPE (30 μM). CAPE treatment induced phosphorylation of ERK, p38, and JNK; whereas, each inhibitor effectively attenuated the phosphorylation of corresponding proteins in HT1376 cells (Fig. 4C–E). The CAPE-induced expression of GDF15, NDRG1, or maspin was repressed by ERK or p38 inhibitors in HT1376 cells (Fig. 4C and E). However, pretreatment with the JNK inhibitor blocked the CAPE-induced maspin but not GDF15 or NDRG1 (Fig. 4D).

### CAPE inhibits tumor growth *in vivo*

**Fig. 5:**
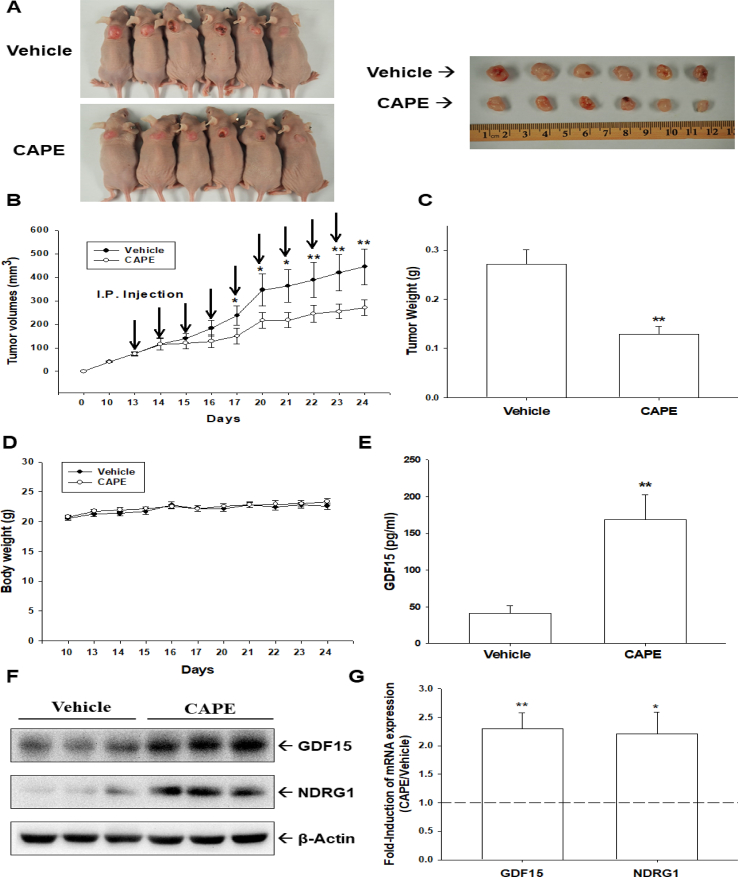
**CAPE inhibits tumor growth of HT1376 cells in xenograft mice model**. The athymic male nude mice were subcutaneously injected with HT1376 cells and when tumor volumes reached around 75 mm^3^ (day 13), they received vehicle (0.1% DMSO in PBS; n = 6) or CAPE (10 mg/kg; n = 6) injected once intraperitoneally per day, 5 days/week. (A) The mice were sacrificed and the tumors were collected and photographed. (B) Tumor volumes were measured in vehicle-treated (●) or CAPE-treated (○) groups. (C) The tumor weights were presented as mean tumor weight in grams. (D) The average body weight of mice was measured during the experimental period. (E) Whole cell lysates of randomly selected tumor samples (n = 3) from the vehicle or CAPE-treated groups were subjected to immunoblotting (F) and RT-qPCR (G) assays. ∗∗*P* < 0.01, ∗*P* < 0.05.

To evaluate the antitumor effect of CAPE in bladder carcinoma cells *in vivo*, HT1376 cells were xenografted. On day 13 after cell inoculation, when the growth of a solid tumor was established by approximately 75 mm^3^, the xenografted mice were randomly divided into two groups (n = 6). CAPE (10 mg/kg/day) or vehicle (1/10,000 DMSO in PBS) were injected intraperitoneally once daily for 5 days per week. After two weeks, the tumors derived from CAPE-treated mice reduced by 53% in size (274.13 ± 17.82 mm^3^ vs. 132.15 ± 18.57 mm^3^) and 48% in tumor weight (0.27 ± 0.03 g vs. 0.13 ± 0.02 g) (Fig. 5A–C). The average bodyweight of the CAPE-treated mice was not significantly (*P* = 0.329) different from vehicle-treated (20.71 ± 0.34 g in the CAPE-treated group vs. 20.86 ± 0.31 g in the vehicle-treated group; Fig. 5D). ELISAs of the serum samples, which were collected from experimental animals by cardiocentesis, revealed that the blood levels of GDF15 in animals injected intraperitoneally with CAPE were 4.1-fold higher than those animals injected with PBS (Fig. 5E). Three tumor tissues of mice were randomly selected from each group for immunoblotting (Fig. 5F) and RT-qPCR (Fig. 5G), confirming that expressions of GDF15 and NDRG1 were significantly (*P* = 0.0094 and *P* = 0.034) higher in the xenograft tumors derived from the CAPE-treated group than those derived from the PBS-treated group. Collectively, CAPE treatment induced GDF15 expression and secretion to attenuate the growth of bladder carcinoma HT1376 cells *in vivo*.

### **AMPK**α**1/2-knockdown attenuates CAPE-induced GDF15 expression and proliferation**

**Fig. 6:**
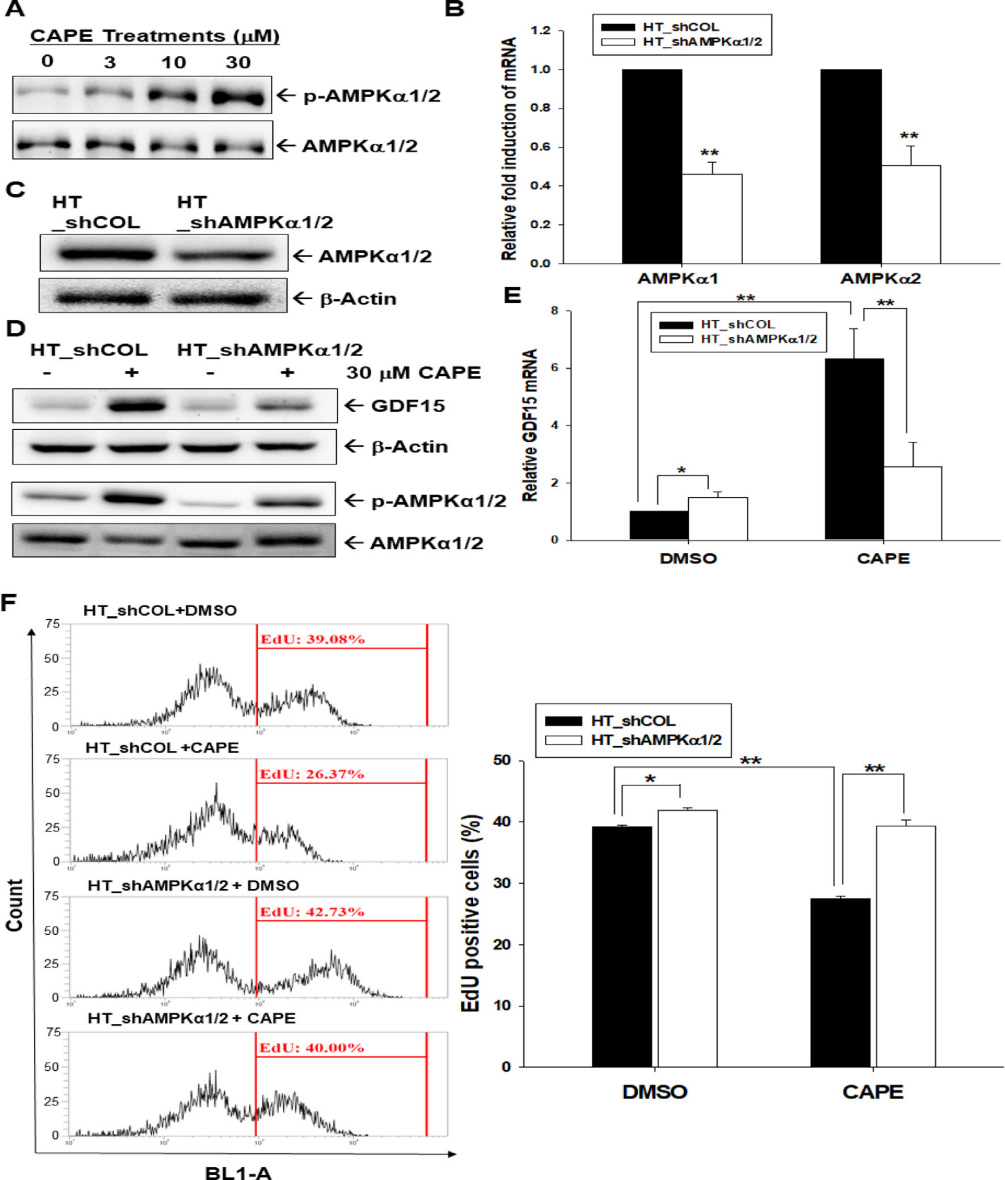
**CAPE upregulates the activities of the AMPKα1/2 signaling pathway in bladder carcinoma cells**. (A) HT1376 cells were treated with various concentrations of CAPE for 24 h, and the protein levels of the AMPKα1/2 and p-AMPKα1/2 were determined using immunoblot assays. (B) The expression of AMPKα1 and AMPKα2 was determined by RT-qPCR in HT_shCOL and HT_shAMPKα1/2 cells (n = 3). (C) The expression of AMPKα1/2 and β-actin was determined by immunoblotting in HT_shCOL and HT_shAMPKα1/2 cells. The protein level of AMPKα1/2 and β-actin was determined by immunoblot assays (D) and the mRNA level of GDF15 was determined by RT-qPCR assays (E; n = 3) in HT_shCOL and HT_shAMPKα1/2 cells after (+) or without (−) 30 μM CAPE treatment. (F) The proliferation of HT_shCOL and HT_shAMPKα1/2 cells was determined by the EdU assays after (+) or without (−) 30 μM CAPE treatment for 24 h (n = 4). ∗∗*P* < 0.01, ∗*P* < 0.05.

Activation of AMPKα1/2 increased in a dose-dependent manner with CAPE treatment (0–30 μM) in HT1376 cells (Fig. 6A), which was observed as early as 20 min after treatment. The transduction of human-specific AMPKα1/2 shRNA downregulated the gene expressions of both AMPKα1 and AMPKα2 determined by RT-qPCR (Fig. 6B) and immunoblot assays (Fig. 6C). Furthermore, knockdown of AMPKα1/2 blocked the effect of CAPE on the phosphorylation of AMPKα1/2 and the expression of GDF15 in HT-1376 cells (Fig. 6D and E), as well as significantly (*P* = 0.0125) upregulated the proliferation of HT1376 cells. CAPE treatment (30 μM) downregulated EdU staining by 13% in TH1376 cells compared to the vehicle control but did not significantly (*P* = 0.917) inhibit cell proliferation when AMPKα1/2 was knocked down in HT1376 cells (Fig. 6F).

## Discussion

GDF15 is involved in cell growth control [4] and is expressed in various cells, including epithelial cells, cardiomyocytes, adipocytes, macrophages, endothelial, and vascular smooth muscle cells [21]. GDF15 has a diverse range of tissue-specific and cell-specific presentations [22,[24], [25], [26]]. Previously, we reported that GDF15 is upregulated *via* DNA demethylation and p53; moreover, GDF15 modulated the gene expressions of NDRG1, maspin, and epithelial–mesenchymal transition (EMT) markers to downregulate cell proliferation, invasion, and growth of bladder carcinoma cells [7]. Since then, several reports have confirmed that GDF15 is a methylation-targeted antitumor marker for bladder cancer [[27], [28], [29]].

Results of the present study verified that both protein and mRNA levels of GDF15 were higher in normal HBdEC and papilla bladder cancer cells (RT-4) compared to other advanced bladder carcinoma cells, supporting that GDF15 expression is negatively correlated with neoplasia *in vitro* from our previous study [7]. The protein of α-SMA, an actin isoform involved in smooth muscle contractility in the human bladder, expressed higher levels in the HBdSMC [30], and the cells of HBdEC exhibited higher protein levels of UPK2, a marker of bladder transitional cells [31], verifying either smooth muscle-specific or transition epithelium-specific characteristics for different bladder cells. This is the first report that the HBdSF cells, either fibroblast or smooth muscle cells, as well as human bladder carcinoma cells secret GDF15 *in vitro.*

Propolis (bee glue) has been widely used in traditional medicine and is regarded as a health supplement. CAPE possesses most of the pharmacological-active components of propolis, with antidiabetic, anti-oxidant, anti-viral, anti-bacterial, anti-inflammatory, and antitumor properties [13]. This study confirmed that CAPE inhibits cell proliferation, invasion, and tumor growth of bladder carcinoma cells via upregulation of GDF15.

Previously, GDF15 has been shown to modulate the gene expressions of NDRG1, maspin, and EMT markers to downregulate proliferation, invasion, and tumor growth of bladder carcinoma cells [7]. Also, several studies have suggested that NDRG1 is a tumor suppressor gene in bladder carcinoma cells [7,32]. In the present study, CAPE treatment upregulated NDRG1 expression, in line with our previous reports regarding OSCC and NPC cells *in vitro* [18,19]. Maspin is a novel tumor suppressor gene in transitional cell carcinoma of the bladder, although it has divergent effects on different types of cancers [33]. Recently, maspin was found to be upregulated by p53 and PTEN, as well as acting as an HDAC1 inhibitor, thereby blocking the proliferation and invasion of human bladder carcinoma cells *in vitro* and *in vivo* [34]. The present study revealed that CAPE induced-expressions of NDRG1 and maspin were dependent on the GDF15. Taken together, CAPE treatment upregulating GDF15 secretion *in vitro* and *in vivo* induced the gene expressions of NDRG1 and maspin in bladder carcinoma cells.

The possible molecular signaling pathways of CAPE involved in cancer development include NFκB, p53, Wnt/β-catenin, PI3K/Akt, and so forth [13]. The MAPK pathways contributed to several diverse cellular activities including mitosis, metabolism, motility, survival, apoptosis and differentiation, in which triggers oncogenic and tumor-suppressor roles [35]. Upregulation of the MAPK pathways has been identified as one of the anti-cancer mechanisms of CAPE in OSCC, NPC, glioma, and prostate carcinoma cells [18,19,36,37]. Our results indicated that CAPE modulates ERK, JNK, and p38 phosphorylation, and the upregulations of GDF15, NDRG1, and maspin by CAPE were attenuated only by pretreatments with inhibitors of ERK (PD0325901) or p38 (SB201290) but not JNK (SP600125). The present study not only verified that CAPE upregulates MAPK signaling pathways but also identified the expression signatures of downstream target genes in bladder carcinoma cells. Our previous studies suggested that CAPE upregulated NDRG1 expression via the p38 signaling pathway in NPC but not in OSCC cells [18,19]. Overall, CAPE upregulates multiple MAPK signaling pathways in bladder carcinoma cells as in OSCC and NPC cells, but the modulation of CAPE in expressions of NDRG1, maspin, and GDF15 occurs via different signals and is cell-dependent.

AMPK, a heterotrimeric protein consisting of α1/2, β1/2, and γ1/2/3-subunits, is a sensor of cellular energy status, and AMPK activity is involved in normal physiology, fibrosis, aging, and cancer [38,39]. An AMPK agonist was reported to have protective effects on tissues from carcinogenesis [40,41]. Recently, AMPKα activity was found to modulate the contractility of rat detrusor muscle [42]; however, the effects of AMPK activity in the neoplasia of the human bladder are still not clearly explored. Our study revealed that CAPE induced AMPKα1/2 phosphorylation in bladder carcinoma cells (Fig. 6A), in line with a previous study which showed that CAPE increased the activation of AMPKα1/2 in microglial cells [43]. The knockdown of AMPKα1/2 inhibited CAPE-induced GDF15 expression and upregulated the proliferation of HT1376 cells, blocking the inhibitory effect of CAPE on the proliferation of HT1376 cells. These results are consistent with a previous report in microglial cells [43], although their study only used one immunoblot assay to conclude that CAPE treatment increased the activation of AMPKα. Taken together, our study confirmed that CAPE induced GDF15 expression and blocked cell proliferation *via* the AMPKα1/2 signaling pathway.

A recent review suggested that the competitive glucose metabolism is the target to enhance the bladder cancer immunotherapeutic effects [44]. Our results are the first study to reveal that CAPE upregulated GDF15, a sensor of systemic metabolism. Whether CAPE suppresses bladder carcinoma cells by reprogramming the metabolic process is still inconclusive. The accurate mechanisms associated with CAPE involved in human bladder cancer still need further investigation. Furthermore, the development of CAPE as an additive agent to enhance the efficacy of intravesical therapy clinically for the bladder cancer is warranted.

## Conclusions

GDF15, an antitumor gene in bladder carcinoma cells, is abundant in stromal, normal epithelial, and papillary bladder cancer cells. CAPE attenuates proliferation, invasion, and growth of bladder carcinoma cells *in vitro* and *in vivo*, inducing the expressions of GDF15, NDRG1, and maspin *via* ERK, p38, or AMPKα1/2 signaling pathways. Collectively, our study suggests that CAPE actives MAPK and AMPK signaling pathways to induce expressions of GDF15 and its target downstream genes, NDRG1 and maspin. CAPE inhibits the growth of human bladder carcinoma cells *in vitro* and *in vivo* indicating that CAPE is a promising preventive agent in the tumor growth of human bladder carcinoma cells.

## Ethics approval and consent to participate

Not applicable.

## Consent for publication

Not applicable.

## Data availability statement

The data used to support the findings of this study are available from the corresponding author upon request.

## Funding

This research was funded by grants from the 10.13039/501100004663Taiwan Ministry of Science and Technology (MOST 110-2314-B-038-151-MY3 and MOST-109-2320-B-182-018-MY3) and 10.13039/100012553Chang Gung Memorial Hospital (CMRPG3K0291, CMRPG3L0331-2, and CMRPD1I0111-3).

## Declaration of competing interest

The authors declare no conflicts of interest.
